# Human wound infections caused by *Neisseria animaloris* and *Neisseria zoodegmatis*, former CDC Group EF-4a and EF-4b

**DOI:** 10.3402/iee.v3i0.20312

**Published:** 2013-08-02

**Authors:** Anna Heydecke, Birgitta Andersson, Torsten Holmdahl, Åsa Melhus

**Affiliations:** 1 Department of Medical Sciences, Section of Clinical Bacteriology, Uppsala University, Uppsala, Sweden; 2 Department of Clinical Microbiology, Malmö University Hospital, Malmö, Sweden; 3 Department of Infectious Diseases, Malmö University Hospital, Malmö, Sweden

**Keywords:** EF-4, N. animaloris, N. zoodegmatis, animal bite, wound infection

## Abstract

**Background:**

*Neisseria animaloris* and *Neisseria zoodegmatis*, former CDC Group EF-4a and -4b, are considered to be rare zoonotic pathogens, usually associated with dog or cat bites. The aim of the study was to phenotypicaly characterize 13 EF-4 isolates from wound infections, determine their antibiotic susceptibility and to follow the clinical outcome of the patients.

**Methods:**

13 of the EF-4 isolates were cultured on agar plates. Conventional biochemical tests and the Biolog system were used for phenotypical identification. An arbitrary primed polymerase chain reaction (AP-PCR) was carried out to determine the genetic profiles. Minimum inhibitory concentration (MIC) values were determined for different antibiotics were determined. According to this, clinical data for the patients were recorded.

**Results:**

11 isolates were identified as *N. animaloris* and 2 as *N. zoodegmatis* due to the production of arginine dihydrolase. A majority of the patients had a history of dog bite. In 6 cases only grewth of *N. animaloris* or zoodegmatis was registered. When a patient received antibiotic treatment the most common drug of choice was penicillin V. Only 3 patients received treatment for which the isolated EF-4 bacterium was fully susceptible.

**Conclusion:**

Human infections involving *N. animaloris* and *N. zoodegmatis* usually present themselves as local wound infection, but severe complications can occur. Despite their pathogenic potentia, l *N. animaloris* and *N. zoodegmatis* are often misidentified, dismissed as skin contaminants or not recognized at all. Due to the fact that *N. animaloris* and *N. zoodegmatis* are significant pathogens in animal bites, physicians should keep these bacteria in mind when choosing antibiotic therapy.

Pets are common in many households worldwide and are a source of bites and scratches. Dogs account for the majority of mammalian bites (1, 2) and approximately 1% of all visits to the emergency room in the United States are due to dog bites (3).

The most common complication following an animal bite is a wound infection, which tends to be polymicrobial and include both aerobic and anaerobic bacteria. Pasteurella spp. predominate in infected dog and cat bites. A less recognized group of bacteria that has been identified as human pathogens following animal bites is the former Center for Disease Control group Eugonic Fermenter-4 (CDC Group EF-4). EF-4 bacteria are part of the normal oral flora of dogs and cats. They are one of the most frequently isolated bacteria from the gingiva and nasal and oral secretions of healthy canines (4, 5), and they may cause pulmonary infections in dogs and cats (6).

Most of the biochemical characteristics of these Gram-negative coccoid or short rods were first described in 1974 by Tatum and co-workers (7), and two biotypes, EF-4a and EF-4b, were recognized. The EF-4a strains fermented glucose and were arginine dihydrolase-positive, whereas EF-4b strains oxidized glucose and were arginine dihydrolase-negative. Both types yielded small yellow or non-pigmented colonies on blood and chocolate agars, were non-motile, oxidase-positive and catalase-positive. They reduced nitrate to nitrite but did not hydrolyze urea or produce indole. Recent studies have shown that the EF-4 group belongs to the genus *Neisseria* and that EF-4a and EF-4b represent two novel species within this genus, *Neisseria animaloris* and *Neisseria zoodegmatis*, respectively (8).

Due to the polymicrobial aspect of infected bite wounds, broad-spectrum antibiotics, such as amoxicillin/clavulanic acid, are often recommended as empiric treatment of animal bites (9). In Scandinavia, penicillin V and oxacillin are often used for treating animal bites. The high prevalence of EF-4 bacteria in the commensal flora of dogs and cats suggests that they might commonly contaminate or cause infections in bite wounds but have so far probably been overlooked. The aim of this study was to investigate 13 not-fully identified isolates from patients with dog- or cat-associated infections, characterize the isolates with conventional methods, and follow the clinical course of the infections to elucidate if overlooking *N. animaloris* and *N. zoodegmatis* might have clinical consequences.

## Materials and methods

### Bacteria and media

A total of 13 isolates were collected during a period of 15 years (1982–1997) at the Department of Clinical Microbiology, Malmö University Hospital, Sweden. For inclusion, the bacteria had to be Gram-negative coccoid rods, oxidase-positive, indole-negative, and yield poor or no results in a conventional Pasteurella test (10).

The isolates were cultured on blood and chocolate agar (Difco Laboratories, Detroit, MI, USA) and a selective medium for *Enterobacteriaceae* (11). The plates were incubated at 37°C in the presence of 5% CO_2_, in ambient air, and anaerobically for up to 7 days.

### Identification and antibiotic susceptibility

To identify the isolates, the following conventional biochemical tests were carried out: production of catalase, urease, decarboxylases (ornithine, lysine), arginine dihydrolase, β-galactosidase (ONPG), β-glucoronidase (PGUA), and β-xylosidase (ONPX); acid production from glucose, lactose, maltose, and saccharose; reduction of nitrate. In addition, the Biolog system (Biolog Inc., Hayward, CA, USA) was used, but not 16S rRNA sequencing or MALDI-TOF MS as these methods are usually only available in clinical microbiology laboratories at tertiary hospitals in Scandinavia. Two randomly selected isolates were sent to the Culture Collection, University of Gothenburg, Sweden (CCUG), for confirmation of the results. The isolates were incubated aerobically, anaerobically, and in CO_2_ to explore the environmental preferences.

The isolates’ antibiotic susceptibility to penicillin G, ampicillin, oxacillin, cefuroxime, tetracycline, trimethoprim/sulfamethoxazole, ciprofloxacin, gentamicin, and imipenem was tested. minimum inhibitory concentration (MIC) values were determined by Etest^®^ according to the recommendations of the Swedish Reference Group for Antibiotics (www.srga.org).

The control strains tested included CDC Group EF-4a ATCC 29858, CDC Group EF-4b ATCC 29859, and *Neisseria weaveri* CCUG 4007.

### DNA fingerprinting

To determine the genetic profiles of the isolates, an arbitrary primed polymerase chain reaction (AP-PCR) was carried out. The DNA was prepared as described by Jackson and Cook (12). Three different primers were used: A70-4, A70-3, and 60-10 (Genosys). The mixture was processed for 45 cycles in a Rapid cycler (Idaho Technology, Idaho, USA) with the following cycling parameters: 2 s of denaturation at 94°C, 30 s of annealing at 40°C, and 30 s of extension at 72°C. The reaction was finally extended for 5 min at 72°C. The isolates were run 2–3 times to assess the reproducibility. An total of 10 µL of the polymerase chain reaction (PCR) product was electrophoresed through a 1.5% agarose gel (Saveen Agarose Standard Molecular Biology Grade, Saveen Biotech AB) with ethidium bromide in Tris-borate-EDTA buffer (pH 8). The DNA bands were visualized under UV light and photographed. The size of the amplified product was compared with a DNA ladder (Life Technologies AB, Boehringer Mannheim, Mannheim, Germany).

### Patient data

The patients’ physicians were contacted and the following patient data were recorded: time elapsed between animal bite and first contact with a physician and prescription of antibiotics, animal that caused the wound, location of the wound, culture results, the type of antibiotic treatment given, need for surgical revision and clinical outcome.

## Results

### Phenotypic characteristics

After 48 h of aerobic incubation, the isolates grew with yellowish-white colonies. CO_2_ did not enhance growth noticeably, and anaerobic incubation yielded poorer growth. None of the isolates grew on the selective medium for *Enterobacteriaceae*.

The isolates were catalase-positive, reduced nitrate, and produced acid from glucose but not from any other sugar. Due to the production of arginine dihydrolase, 11 isolates were identified as *N. animaloris* and 2 as *N. zoodegmatis*. The remaining biochemical tests were all negative.

Preferred carbon sources in the Biolog System were d,l-lactic acid (13/13 isolates), acetic acid (12/13), methyl pyruvate (12/13), glycyl-l-glutamic acid (11/13), α-d-glucose (10/13), l-glutamic acid (10/13), succinate acid (9/13), and α-butyric acid (8/13). The carbon sources were similar for the two species. The two strains (one arginine-positive and one arginine-negative), which were sent to CCUG for confirmation, were identified as *N. animaloris* and *N. zoodegmatis*, respectively.

### Antibiotic susceptibility

The antibiotic susceptibility testing showed that *N. animaloris* was the most resistant species of the two (Table 1). The *in vitro* activity for ampicillin, imipenem, and ciprofloxacin was high, but otherwise there were few treatment options for this bacterium. When using non-species-related breakpoints, eight of the *N. animaloris* isolates were resistant to both cefuroxime and gentamicin but none of the *N. zoodegmatis* isolates. As expected, it was not possible to treat any of the 13 isolates with isoxazolyl penicillin.

**Table 1:** MICs of the *N. animaloris* and *N. zoodegmatis* isolates included in the study

	MIC (mg/L)								
	
Case no.	PG *S≤0.25, *R*>2	AM *S*≤2, *R*>8	OX –	XM *S*≤4, *R*>8	IP *S*≤2, *R*>2	TS –	TC –	GM *S*≤2, *R*>4	CI *S*≤0.5, *R*>1
1	1	1	>256	16	0.5	0.5	1	8	0.016
2	4	1	>256	32	0.25	0.25	2	8	0.016
3	4	1	>256	32	0.5	0.5	1	8	0.016
4	2	0.5	>256	8	0.5	0.25	2	4	0.016
5	1	0.5	>256	8	0.25	0.5	2	4	0.016
6	1	0.5	>256	8	0.25	0.25	2	4	0.016
7	4	1	>256	16	0.25	2	1	8	0.032
8**	***	0.032	8	0.125	0.064	0.064	0.5	0.5	<0.002
9	4	1	256	64	0.25	1	0.5	8	0.032
10	1	0.5	64	16	0.125	0.5	0.25	8	0.032
11	4	0.5	>256	32	0.25	1	2	16	0.032
12**	0.25	0.25	32	0.5	0.125	0.125	0.25	2	0.002
13	1	0.5	256	16	0.25	0.25	1	8	0.016

* PG=penicillin G; AM=ampicillin; OX=oxacillin; XM=cefuroxime; IP=imipenem; TS=trimethoprim-sulfamethoxazole; TC=tetracycline; GM=gentamicin; CI=ciprofloxacin. Non-species-related breakpoints are given below.

** *N. zoodegmatis*. In all other cases, *N. animaloris* was isolated.

*** MIC missing, recorded as susceptible to penicillin G in laboratory records.

### Clinical data

Of the 13 patients, 7 were male and 6 were female with a median age of 56 years (range 5–79 years). Eleven patients had dog bite wounds. A majority of the wounds were localized to the hands (hand *n*=7; arm/shoulder *n*=1; leg *n*=2, face *n*=1; leg and arm *n*= 1). Data on the causative animal were missing for two patients and the localization for one patient.

About half of the patients visited a physician on the day they received the bite, whereas the others waited 1–21 days (median 2 days) before they made the visit. In most cases, the patients were diagnosed with a wound infection, but one patient developed septicemia and another tenosynovitis with a tendon rupture. One patient had a tumour in the wound area. Four of the patients were admitted to the hospital. Of the hospitalized patients, three had been bitten on the hand and one on the arm/shoulder.

In six cases, only growth of *N. animaloris* or *N. zoodegmatis* was registered (Table 2). Ten patients were given one or more antibiotics within 7 days after the bite. The most common choice was penicillin V for 10 days. Two patients required intravenous treatment. In contrast, two patients did not receive any antibiotic drug at all. Only three patients received treatment for which the isolated EF-4 bacterium was fully susceptible. Apart from the antibiotic treatment, four patients required open drainage or surgical revision of the wound. Three patients were vaccinated against tetanus. Follow-up data were available for all patients but one. On average, it took at least 14 days for the patients to recover (Table 2).

**Table 2:** Therapy and outcome of 13 patients in relation to microorganisms

Case no.	Organisms isolated	Delay before contact (days)	Antibiotic treatment	Clinical outcome
1	*N. animaloris*	0	Penicillin V	Not fully recovered on day 9 (last visit)
2	*N. animaloris, Staphylococcus epidermidis*	2	None	Not fully recovered on day 13 (last visit)
3	*N. animaloris*, *Pasteurella multocida*	0	None	Not fully recovered on day 14 (last visit)
4	*N. animaloris*	0	Cefadroxil	Wound deterioration during therapy. Full recovery after 24 days
5	*N. animaloris*	21	Data missing	Data missing
6	*N. animaloris, Staphylococcus aureus, group B streptococci*	Data missing	Cefuroxime, cephalexin	Hospitalized, tumor discovered, surgically removed and radiated
7	*N. animaloris, P. multocida, coagulase-negative staphylococci*	0	Penicillin V	Hospitalized 2 weeks, not fully recovered after 1.5 months, missed last visit
8	*N. zoodegmatis*	2–3	Flucloxacillin, erythromycin	Hospitalized, not fully recovered at last visit after 1.5 months. Tendon rupture with a loss of flexion in a finger. Referred to other clinic
9	*N. animaloris*	1	Penicillin V, cefadroxil	Not fully recovered on day 15. Referred to hand surgeon due to fracture
10	*N. animaloris, P. multocida*, anaerobes, streptococci	1	Flucloxacillin, penicillin V	Full recovery on day 14
11	*N. animaloris*, *Enterobacter cloacae*	7	Klindamycin, ciprofloxacin	Not fully recovered on day 28 (last visit)
12	*N. zoodegmatis*	1	Cloxacillin, ampicillin	Hospitalized due to septicemia, full recovery after 2 months
13	*N. animaloris*, enterobacteria	0	Penicillin V, ciprofloxacin	Full recovery after 2 months

### Genetic fingerprinting

**Fig. 1:**
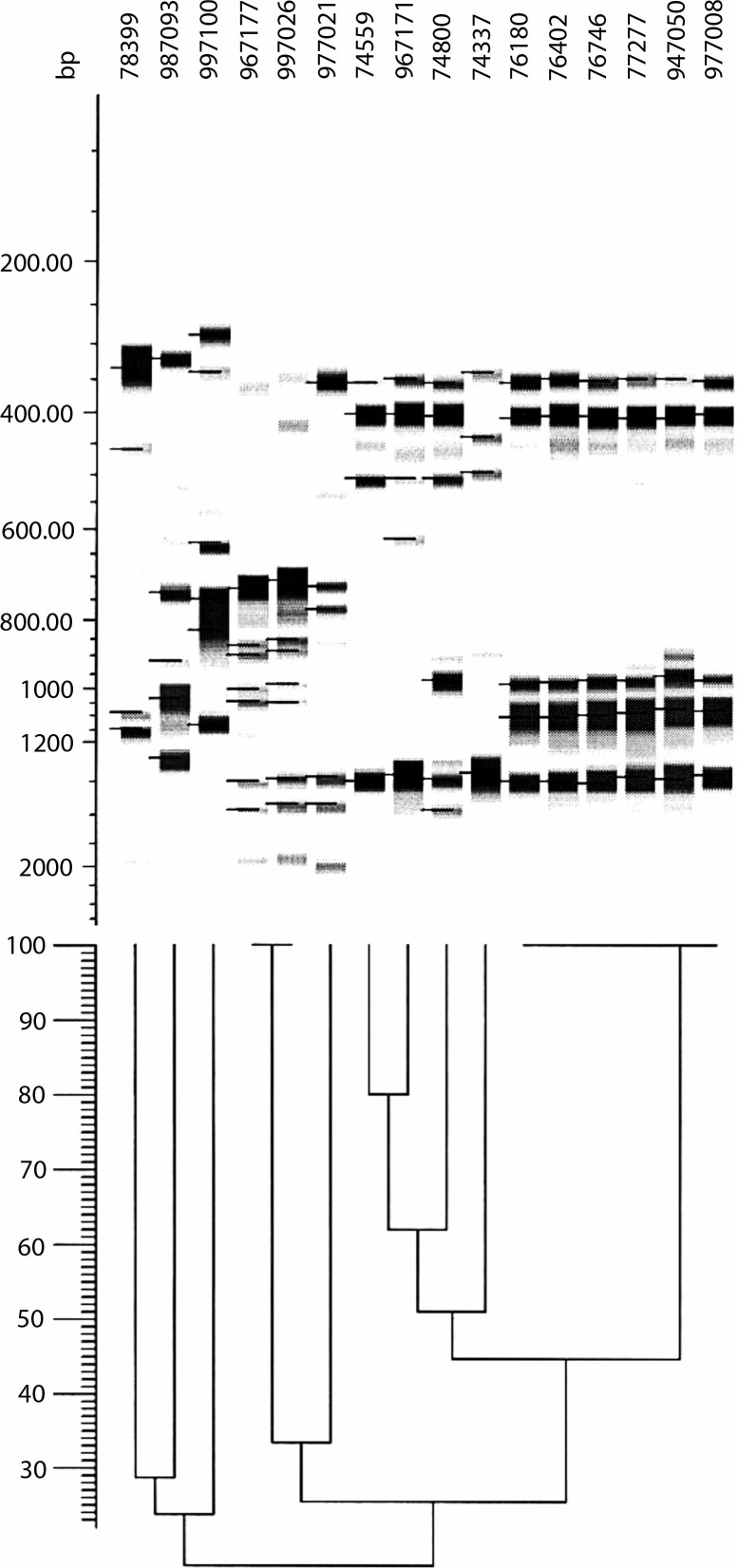
Dendrogram showing the result of an AP-PCR run with primer A70-3. Isolates 78399, 987093, 997100, 997026, and 977021 were identified as *N. zoodegmatis* and the rest as *N. animaloris*.

The two bacterial species exhibited two different DNA profiles: *N. animaloris* was relatively homogeneous. Despite several years passing in between the isolation of the *N. animaloris* isolates, six of them still exhibited identical or highly similar band patterns with the different AP-PCR primers (see Fig. 1). In contrast, the two *N. zoodegmatis* isolates had less than 30% of the bands in common, and that was independent of primer. As there were so few *N. zoodegmatis* isolates, three more isolates from later years were added (987093, 997026, 997100). This addition did not alter the picture.

## Discussion

In this study, 13 not-fully identified Gram-negative coccoid rods isolated mainly from dog bites were characterized. Based on carbon sources used, biochemical reactions and confirmation at CCUG, the isolates were identified as *N. animaloris* and *N. zoodegmatis*. This is, to our knowledge, the largest study dealing with human infections caused by these two bacterial species so far.

Group EF-4 bacteria are considered to be commensals of the oral cavity in dogs, cats, and rodents, but they can also cause severe infections, that is pulmonary and purulent cutaneous infections, in the host animals. In humans, the organisms have been associated with dog and cat bites, but in some instances, they have induced infections merely after close animal contact (13).

Human infections involving *N. animaloris* and *N. zoodegmatis* usually present themselves as local wound infections. Group EF-4 bacteria have, however, been reported to cause chronic otitis media (13), bacteremia (14, 15), and endophtalmitis (16). In our study, local wound infections after dog bites predominated, but there were two patients who developed complications in the form of septicemia and tenosynovitis with tendon rupture, respectively. The pathogenic capacity was further shown by the fact that growth of group EF-4 bacteria was the only bacterial finding in six of the patients. Despite their pathogenic potential, *N. animaloris* and *N. zoodegmatis* are often misidentified as *Pasteurella* spp. or dismissed as skin contaminants. Sometimes they are not recognized at all, which is not a result of poor growth but rather of difficulties with the identification. *N. animaloris* and *N. zoodegmatis* grew promptly on both blood and chocolate agar after 2 days of incubation in ambient air, which shows that they can grow in any routine culture from a wound. The yellowish-white colony color combined with a negative spot indole test made them easy to differentiate from *Pasteurella multocida*, another common bacterium in animal bites. For final identification, the Biolog system and production of arginine dihydrolase were used. This system made it possible to identify group EF-4 bacteria in a clinical microbiology laboratory without using molecular techniques or MALDI-TOF. An instrument, like Vitek 2 from bioMérieux, Inc. (17), could serve as a possible alternative to the Biolog system, but the API^®^ and the Remel system RapID™ do not cover these organisms. Once the staff had learnt how to identify group EF-4 bacteria, the strain collection of *N. animaloris* and *N. zoodegmatis* grew rapidly and it became obvious that these bacterial species were not as rare as assumed earlier.

Few previous studies have reported on the prevalence of EF-4a and EF-4b in animal bite wounds, but there are reports that suggest that EF-4a or *N. animaloris* is most common in human as well as in animal infections (18–20). This is in accordance with the findings in this study, in which 85% of the isolates were *N. animaloris*. However, *N. zoodegmatis* appears to be the most frequent species in the gingival flora of dogs (21). The reason for these divergent findings is unclear, but it has been suggested that *N. animaloris* may be more virulent and thereby more prone to cause infections. The fact that *N. zoodegmatis* was isolated from the two patients with the most severe infections may therefore seem contradictory, but it is more than likely that the outcome of these infections was more dependent on the severity of the bites, the subsequent surgical revisions and inadequate antibiotic treatment than the bacterial species *per se*.

One of the more striking findings when going through the clinical data was the lack of proper antibiotic treatment of the patients. The awareness of common pathogens in dog bites appeared to be low. As shown in this article, not even when there was a documented deterioration of the wound during cefadroxil treatment and a positive culture with an antibiogram, did the prescriber change antibiotics. There is evidently room for improvement in this field, and the use of amoxicillin should be increased when infections with *Neisseria spp*. from dogs or cats are suspected. This is also in line with the current recommendations from the latest workshop arranged by the Swedish Medical Products Agency: amoxicillin with clavulanic acid is the drug of choice for animal bites. The clavulanic acid has been added to include *Stapylococcus aureus* in the treatment. However, *S. aureus* found in animal bite wounds is often *Staphylococcus pseudintermedius*, a bacterium carried by about 50% of the dogs (22). Most Swedish laboratories on the human side cannot differentiate between the two species, and the β-lactamase production is not as frequent in *S. pseudintermedius* as in *S. aureus* (22). A recent review of the bacterial findings in animal bites during the last year at the clinical microbiology laboratory at Uppsala University Hospital, showed that *S. aureus*/*S. pseudintermedius* was only found in 12% of the bites whereas EF-4 or EF-4-like bacteria were more than twice as common (unpublished data).

In conclusion, group EF-4 bacteria are significant pathogens in animal bites, and their isolation frequency in bite wounds is possibly underestimated. Given this, physicians should keep these bacteria in mind when choosing antibiotic therapy and preferably use amoxicillin with or without clavulanic acid instead of penicillin V, flucloxacillin, or cefadroxil.
